# Neuromuscular Complications With SARS-COV-2 Infection: A Review

**DOI:** 10.3389/fneur.2020.01052

**Published:** 2020-09-17

**Authors:** Nakul Katyal, Naureen Narula, Sudeep Acharya, Raghav Govindarajan

**Affiliations:** ^1^Department of Neurology, University of Missouri, Columbia, MO, United States; ^2^Department of Pulmonary- Critical Care Medicine, Staten Island University Hospital, New York, NY, United States; ^3^Department of Internal Medicine, Staten Island University Hospital, New York, NY, United States

**Keywords:** COVID 19, SARS-CoV-2, neuromuscular, neurology, complications, pathophysiology

## Abstract

Severe acute respiratory syndrome coronavirus 2 (SARS-CoV-2) cases were first reported in Wuhan, Hubei province of China in December, 2019. SARS- COV-2 primarily affects the cardio-respiratory system. Over the last few months, several studies have described various neurological sequelae of SARS-COV-2 infection. Neurological complications are more frequent in patients with severe respiratory infections. In this review, we have analyzed the current literature on neuromuscular complications associated with SARS-COV-2 and highlighted possible mechanisms of neuromuscular invasion. We reviewed 11 studies describing 11 cases of Guillain Barre syndrome (GBS), and 1 case each of Miller Fisher syndrome, Polyneuritis Cranialis, Acute myelitis, Oculomotor paralysis and Bell's Palsy associated with SARS-COV-2 infection. Mean age of patients with GBS was 61.54 years, with standard deviation (SD) 14.18 years. Majority patients had fever and cough as the first symptom of SARS COV-2 infection. Mean time for onset of neurological symptoms from initial symptoms in 11 patients was 8.18 days, with SD of 2.86 days. Mean time to performing electrodiagnostic study from onset of neurological symptom was 6 days with standard deviation of 3.25. Six patients had demyelinating pattern, three had acute sensory motor axonal neuropathy, and one had acute motor axonal neuropathy on electrodiagnostic studies.

## Introduction

In December 2019, several reports of patients with severe pneumonia of unknown causes emerged from Wuhan, Hubei province of China (1). In February 2020, the International Committee on Taxonomy of Viruses officially renamed the novel coronavirus responsible for this outbreak as severe acute respiratory syndrome coronavirus 2 (SARS-CoV-2) (2). The World Health Organization declared SARS- COV-2 as pandemic on March 11, 2020 (3). Since then, the epicenter of the pandemic has moved from China to Europe then to North America and Asia.

The first neurological complication from SARS-CoV-2 was reported as a case of viral encephalitis on March 4, 2020, at Beijing Ditan Hospital (4). In a retrospective case series of 214 patients with SARS-COV-2 infection from Wuhan, neurologic symptoms were seen in 36.4% of patients and were more common in patients with severe respiratory infections; these included acute cerebrovascular events, impaired consciousness, and muscle injury (5).

Neuromuscular complications such as critical illness myopathy, polyneuropathy, and guillain barre syndrome (GBS) have been reported with prior SARS outbreaks of 2003 and Middle East respiratory syndrome (MERS) (6, 7). Over the last few months, several studies have described numerous neuromuscular complications in patients with SARS-COV-2 infection. This article presents a narrative review of the current literature on neuromuscular complications associated with SARS- COV 2 infection and describes the possible underlying mechanism of neuro-muscular invasion.

## Method

### Search Strategy and Selection Criteria

We searched Medline, Google Scholar, and Pubmed using keywords; “Neurological,” “Neurology,” “Neuromuscular,” “complications,” “SARS COV-2,” “COVID-19.” Search was limited to English language manuscript only. The literature search was last done on 31st May, 2020. At the time of writing this article, we identified 53 research literature describing neurological complications in SARS-COV-2, out of these, 11 described neuromuscular complications in SARS- COV-2 (8–18). Out of these 11, seven studies described 11 cases of GBS, one described Miller Fisher syndrome and Polyneuritis Cranialis, one described Acute myelitis, one described Oculomotor paralysis and 1 described Bell's Palsy (8–18).

Table 1 describes the demographic data, time to onset of neurological symptoms, diagnostic criteria, intervention and outcomes from 11 reported studies with neuromuscular complications associated with SARS- COV-2 infection.

**Table 1:** Description of demographic data, time to onset of neurological symptoms, diagnostic criteria, intervention and outcomes from 11 reported studies with neuromuscular complications associated with SARS- COV-2 infection.

**Number of Patients**	**Sex**	**Age**	**Pre- neuro symptoms**	**Presenting neurological symptoms**	**Time to onset of neuro- symptoms from initial symptom**	**Diagnosis**	**Lumbar Puncture**	**Radiological findings**	**Electrodiagnostic studies**	**Intervention**	**Outcome**	**Time to outcome from onset of neurological symptoms**	**Follow up**	**References**
1	M	71	Low grade fever	Paresthesia, weakness	1 week	GBS	Albumino-cytological disproportion (protein 54 mg/dl, cells: 9 /Ul)	CT head: negative	Decreased to absent SNAP, Markedly increased CMAP distal latency.	Lopinavir ritonavir IVIG (0.4 g/kg/d for 5days)	Death: Severe respiratory failure.	3 days	NA	(8)
1	M	64	Fever, cough	Paresthesia,tetraparesis	11 days	GBS	Albuminocytologic dissociation (protein 1.66 g/l, Cells: normal)	NA	Decreased velocities, absent F waves	Lopinavir +Ritonavir + IVIG (0.4 g/kg/d for 5 days)	Unavailable	NA	NA	(9)
1	M	70	Myalgia, fatigue, cough	Paresthesia, allodynia, flaccid paralysis	10 days	GBS	Albumino-cytologic dissociation	MRI-ruled out myelopathy	Sensorimotor demyelinating polyneuropathy with sural sparing pattern.	IVIG (0.4 g/kg/d for 5 days)	Rehabilitation	15 days	NA	(10)
1	M	54	Fever, cough	Numbness, weakness lower extremities	10 day	GBS	Not Performed	MRI: Unremarkable	Not Performed	IVIG + Hydroxychloroquine	Rehabilitation	Unavailable	NA	(11)
1	F	61	Fatigue	Bilateral lower extremity weakness	1 day	GBS	Albumino-cytologic dissociation (Protein 124 mg/dl, normal cells)	NA	Delayed distal latencies and absent F waves	Umifenovir+ Lopinavir+ Ritanovir+ IVIG	Complete neurological recovery	30 days	NA	(12)
1	M	65	Fever, cough, dyspnea	Bilateral lower extremity weakness	10 days	GBS	Not obtained	MRI Brain, Spine: Negative	Absent SNAP, Decreased CMAP amplitude	Lopinavir + Ritonavir Hydroxychloroquine IVIG(0.4 g/kg/d for 5 days)	Unavailable	NA	NA	(13)
5	F	77	Fever, cough	1.Flaccid areflexic tetraplegia ascending to facial weakness and respiratory failure	7 days	GBS	Albumino-cytologic dissociation (protein:101 mg/dl, cells: 4/UL)	MRI head: Normal. MRI Spine: Enhancement of caudal nerve roots	Decreased ulnar SNAP, decreased tibial and ulnar CMAP. Absent ulnar and tibial F waves.	IVIG 2 cycles	Poor Outcome: Persistent severe UE weakness, dysphagia and LE paraparesis, neuromuscular respiratory failure.	2 weeks	NA	(14)
	M	23	Fever, sore throat	2. Facial diplegia, lower limb paresthesia, ataxia	10 days	GBS	Albumino-cytologic dissociation (protein:123 mg/dl, no cell)	MRI head: Enhancement of b/l facial nerves. MRI Spine: normal.	Decreased ulnar SNAP, decreased tibial and ulnar CMAP. Decrease in facial nerve cMAP amplitude. Absent F waves	IVIG	Decreased facial and extremity weakness	Unavailable	NA	(14)
	M	55	Fever, cough	3. Flaccid tetraparesis, facial weakness.	10 days	GBS	Alb minocytologic dissociation (protein: 193 mg/dl, no cells)	MRI head: Normal. MRI Spine: Enhancement of caudal nerve roots	Decreased ulnar and tibial CMAP Absent ulnar and tibial F waves	IVIG 2 cycles	Poor Outcome: Neuromuscular respiratory failure	1 month	NA	(14)
	M	76	Dry cough, anosmia	4. Flaccidtetraparesis and ataxia	5 days	GBS	Normal protein, no cells	MRI head: normal. MRI Spine: normal	Increased tibial latencies, decreased CMAP amplitude, decreased velocities. Decreased ulnar amplitude.	IVIG	Mild Improvement, Unable to stand at 1 month	1 month	NA	(14)
	M	61	Dry cough, Anosmia, ageusia	5. Facial weakness, flaccid paraplegia,respiratory failure	7 days	GBS	Albuminocytologic dissociation (protein: 40 mg/dl, cells: 3/UL)	MRI head: NA. MRI Spine: normal	Increased tibial latencies, decreased CMAP amplitude, decreased velocities. Decreased sural SNAP, absent tibial F waves.	IVIG + PLEX	Tetraplegic, neuromuscular respiratory failure	1 month	NA	(14)
2	M	50	Fever, cough, malaise, headache	Anosmia, ageusia, right internuclear ophthalmoparesis, right fascicular oculomotor palsy, ataxia	5 day	MillerFisher syndrome	Albuminocytologic dissociation, positive GD1b-IgG antibodies.	NA	NA	IVIG	Full neurological recovery, except residual ageusia and anosmia	2 weeks		(15)
	M	39	Fever, diarrhea	Ageusia, bilateral abducens palsy, areflexia	3 day	Polyneuritis cranialis	Albuminocytologic dissociation	NA	NA	Acetaminophen	Full neurological recovery	2weeks	After 2 weeks, complete recovery	(15)
1	M	64	Fever, fatigue	Flaccid LE paralysis, Urinary and bowel incontinence	7 days	Acutemyelitis	NA	NA	NA	Moxifloxacin + tamiflu + Ganciclovir+Lopinavir+ Ritonavir+Dexamethasone+ IVIG	Rehabilitation	14days	NA	(16)
1	M	62	Weakness	Diplopia, left eye ptosis	5 day	Oculomotor Palsy	NA	MRI, MRA negative	NA	Moxifloxacin, Tamiflu, Lopinavir, Ribavirin, Steroids, IVIG (0.4 g/kg once every day)	Death due to respiratory failure	12 days	NA	(17)
1	F	65	Pain in mastoid region	Left peripheral facial palsy	1 day	Left Bell's Palsy	NA	MRI Brian: Unremarkable	NA	arbidol and ribavirin	Recovery	Unavailable	1month	(18)

## Neuromuscular Complications With SARS-COV-2

### Guillain Barre Syndrome (GBS)

#### Demographics

Seven studies have described a total of 11 cases of GBS (8–14). Out of 11 patients, nine were male, and two was female (8–14). Mean age of these 11 patients was 61.54 years, with standard deviation (SD) 14.18 years. Seven out of 11 patients had fever as the first symptom of SARS COV-2 infection (8–14). Six out of those seven had cough as an accompanying symptom (8–14). Two out of 11 patients had fatigue and myalgia as the first symptom of SARS COV-2 infection (8–14). Remaining two out of 11 patients had cough and anosmia as the first symptom of SARS COV-2 infection (8–14).

Mean time for onset of neurological symptoms from initial symptoms in 11 patients was eight. 18 days, with SD of 2.86 days.

#### Lumbar Puncture

Out of 11 patients, nine underwent lumbar puncture out of which 8 showed albuminocytological disproportion on cerebrospinal fluid analysis. One showed normal protein and no cells. Lumbar puncture was not performed in two patients.

#### Imaging

Imaging studies including computed tomography (CT) head scan, Magnetic resonance imaging (MRI) brain and spine were obtained in nine out of 11 patients. Two patients had MRI evidence of caudal nerve root enhancement on MRI spine, one patient had bilateral facial nerve enhancement. In the remaining six patients, imaging studies were unremarkable.

#### Electrodiagnostic Studies

Electrodiagnostic studies were obtained in 10 out of 11 patients. Mean time to performing electrodiagnostic study from onset of neurological symptom was 6 days with standard deviation of 3.25. Six out of 10 patients had demyelinating patterns (prolonged motor latencies, severe conduction velocity slowing, and conduction blocks) (8–14). Three patients had acute sensory motor axonal neuropathy and remaining one had acute motor axonal neuropathy (8–14). None of the patients had follow up electrodiagnostic study.

Table 2 describes details of electrodiagnostic studies from 10 reported cases of Guillain Barre syndrome associated with SARS- COV-2 infection.

**Table 2:** Description of electrodiagnostic studies from 10 reported cases of Guillain Barre syndrome associated with SARS- COV-2 infection.

**Timing of electrodiagnostic studies from onset of neurological symptoms**	**Electrodiagnostic studies**	**Interpretation**	**Follow up electrodiagnostic studies**	**References**
Day 4	1. Absence of both the sural nerve sensory nerve action potential (SNAP) and the tibial nerve compound muscle action potential (CMAP). 2. Markedly increased common peroneal CMAP distal latency, markedly decreased velocity, moderately decreased CMAP amplitude (with spatial and temporal dispersion) for the same nerve. 3. Decreased ulnar SNAP amplitude.	Acute polyradiculoneuritis, confirmed demyelination	None, Patient died unfortunately.	(8)
Day 5	1. Decreased right median, and bilateral ulnar velocities and increased F wave latencies in same nerves 2. Increased distal latencies and decreased velocities along bilateral peroneal and tibial nerves.	Acute inflammatory demyelinating polyneuropathy.	Unavailable	(9)
Day 5	1. Sensorimotor demyelinating polyneuropathy with sural sparing pattern. 2. F wave study showed decreased persistence or absent F-waves in tested nerves.	Acute inflammatory demyelinating polyneuropathy.	Unavailable	(10)
Day 5	1. Increased left median distal latencies. 2. Increased left ulnar distal latencies and absent F waves 3. Increased bilateral tibial distal latencies and absent F waves	Acute inflammatory demyelinating polyneuropathy.	Unavailable	(12)
Day 9	1.Absent SNAP, Decreased CMAP amplitude along bilateral tibial and median nerves	Acute sensory motor axonal neuropathy	Unavailable	(13)
Day 3	1.Decreased ulnar SNAP, decreased tibial and ulnar CMAP. 2. Absent ulnar and tibial F waves.	Acute sensory motor axonal neuropathy	Unavailable	(14)
Day 12	1. Decreased ulnar SNAP, decreased tibial and ulnar CMAP. 2.Decrease in facial nerve cMAP amplitude. 3. Absent tibial F waves	Acute sensory motor axonal neuropathy	Unavailable	(14)
Day 11	1.Decreased ulnar and tibial CMAP 2. Absent ulnar and tibial F waves	Acute motor axonal neuropathy	Unavailable	(14)
Day 2	1. Increased tibial latencies, decreased CMAP amplitude, decreased velocities. 2. Decreased ulnar amplitude.	Acute inflammatory demyelinating polyneuropathy.	Unavailable	(14)
Day 4	1. Increased tibial latencies, decreased CMAP amplitude, decreased velocities, conduction block. 2. Decreased sural SNAP, absent tibial F waves	Acute inflammatory demyelinating polyneuropathy.	Unavailable	(14)

#### Interventions

All 11 patients received IVIG treatment in combination with various antivirals, antibiotics, and immunosuppressive agents. Majority cases used a standard dose of IVIG; 0.4 mg/kg/day for 5 days. None of the studies mentioned any complications associated with IVIG therapy.

#### Outcomes

Death was reported as an outcome in one of 11 patients. Final outcomes were unavailable/not reports for three patients. Poor outcome defined as persistent/ worsening of symptoms was reported in two patients. One had complete neurological recovery. Remaining four patients had improvement of symptoms with decreased weakness. Though none of the studies mentioned dysautonomia, one patient had hemodynamic disturbances with severe drug-resistant hypertension, suggesting possible autonomic nervous system involvement (8). Four out of 11 patients developed neuromuscular respiratory failure. One patient developed respiratory failure, 3 days from onset of neurological symptoms, one at 2 weeks days from onset of neurological symptoms and remaining two at a month after onset of neurological symptoms.

### Miller Fisher Syndrome

Ortiz et al. reported the first case of Miller fisher syndrome in a 50-year-old man who presented with anosmia, ageusia, right internuclear ophthalmoparesis, right fascicular oculomotor palsy, ataxia, areflexia, 5 days after developing cough, malaise, headache, low back pain, and fever (15). Workup was remarkable for albuminocytologic dissociation and positive testing for GD1b-IgG antibodies (15). SARS- COV-2 infection was confirmed by qualitative real-time reverse-transcriptase–polymerase-chain-reaction assay utilizing oropharyngeal swab (15). The patient was successfully treated with IVIG and achieved complete neurological recovery after 2 weeks, with exception of residual ageusia and anosmia (15).

### Polyneuritis Cranialis

Ortiz et al. also described the first case of polyneuritis cranialis in a 39-year-old male who presented with ageusia, bilateral abducens palsy, areflexia, 3 days after developing diarrhea, a low-grade fever, and a poor general condition (15). Workup was remarkable for albuminocytologic dissociation (15). SARS- COV-2 infection was confirmed by qualitative real-time reverse-transcriptase–polymerase-chain-reaction assay utilizing oropharyngeal swab (15). The patient had normal respiratory, cardiovascular and abdominal examination and therefore was treated symptomatically with acetaminophen only which resulted in full neurological recovery after 2 weeks (15). This case indicates that patients with normal cardio-respiratory exam may have better neurological outcomes.

### Acute Myelitis

Zhao et al. reported the first case of acute myelitis in a 66 year old male who developed flaccid weakness of bilateral lower extremity with bowel and bladder incontinence and sensory level at T 10, 7 days after developing fever (16). SARS- COV-2 infection was confirmed by nucleic testing utilizing nasopharyngeal swab (16). Lumbar puncture and MRI studies were not performed given pandemic related reasons (16). The patient was treated with a combination of moxifloxacin, tamiflu, ganciclovir, lopinavir, ritonavir, dexamethasone, and IVIG (15 g once daily × 7 days) (16). The patient achieved improvement in bilateral upper and lower extremity strength and was eventually discharged to a rehabilitation facility (16).

### Oculomotor Paralysis

Wei et al. described the first case of oculomotor paralysis in a 65 year old male who presented with 5 days history of persistent diplopia, and left eyelid droop (17). The patient had complete ptosis of left eye and the left eye was down and out at rest (17). MRI and Magnetic resonance angiography (MRA) were unremarkable however, CT chest showed diffuse ground glass opacities (17). SARS- COV-2 was detected in the throat swab (17). The patient was treated with a combination of moxifloxacin, tamiflu, ribavirin, lopinavir, methylprednisolone, and IVIG (0.4 g/kg once every day). Unfortunately, he developed respiratory failure and died on day 12 of admission (17).

### Bell's Palsy

Wan et al. described the first case of Bell's Palsy in a 65 year old female who presented with left lower motor neuron facial paralysis, 2 days after developing pain in the mastoid region (18). Interestingly, this patient had no other symptoms of viral illness. MRI brain showed no abnormality however, computed tomography (CT) chest showed patchy areas of ground-glass shadows in the right lower lung raising suspicion for SARS-COV 2 (18). SARS- COV-2 infection was confirmed by real-time reverse-transcriptase–polymerase-chain-reaction assay utilizing throat swabs (18). The patient was successfully treated with arbidol and ribavirin and achieved resolution of neurological symptoms and lung shadows after 1 month (18).

## Mechanism Of Neuromuscular Involvement in SARS-COV-2

### Angiotensin-Converting Enzyme 2 Mediated Pathway

The first major target of SARS- COV-2 is the ACE-2 receptor located on epithelial cells of the respiratory tract (19). This binding results in downregulation of ACE-2 expression as well as the viral entry and replication (20). Loss of ACE-2 expression leads to dysregulation of the renin-angiotensin system which causes an elevated production of angiotensin II (21). The overproduction of angiotensin II results in a cascade of interactions that eventually leads to severe acute lung injury (21).

ACE2 receptors are present widespread throughout the brain, including cardio-respiratory neurons of the brainstem (dorsal vagal complex), endothelial cells, glial cells, basal ganglia, motor cortex, and raphe (21–23). Once in blood circulation, SARS-COV-2 can travel via hematogenous route to infect the endothelial cells of the blood–brain barrier and then accumulate in ACE-2 rich brain regions causing neurological sequelae (21, 24). Respiratory distress experienced during SAR-CoV2 infection may result from compromise of the brainstem's cardiorespiratory center (21, 25, 26).

### Olfactory Pathway

The anatomical organization of olfactory nerve and olfactory bulb in the nasal cavity provides a direct portal for entry of SARS-COV-2 from periphery to CNS (21, 27). After infecting nasal cells, COV can reach the brain and cerebrospinal fluid through the olfactory nerve and olfactory bulb within 7 days and cause inflammation and demyelinating reaction (4).

### Trans-Synaptic Pathway

The major mechanism by which viruses cause neuromuscular complications involves the entry through peripheral nerve endings located in the skin and mucosa (28, 29). This process is followed by an endogenous neuronal mechanism causing retrograde axonal transport of viruses from the cell periphery to the neuronal cell body (28, 29). Multiple other COV-viruses are known to exhibit transsynaptic transfer properties including HCoV-OC43, HEV 67N (21, 30–32). HEV 67N shares more than 91% homology with the novel SARS-CoV-2 thus further consolidating the hypothesis of retrograde transfer as a possible mechanism of neuro-muscular invasion in SARS-COV-2 (21, 30–32).

### Immune Mediated Pathway

Cytokine storm is an immune-mediated life-threatening disease, which is caused by impaired natural killer and cytotoxic T-cell function (33, 34). Viral infection is the most frequent trigger, either as a primary infection in healthy people or after reactivation in immunosuppressed patients (33, 34). Cytokine storm is associated with an exaggerated inflammatory response caused by hypersecretion of proinflammatory cytokines such as interferon γ, tumor necrosis factor α (TNFα), interleukin 1, 4, 6, 8, 10, and 18 which causes tissue damage and progressive systemic organ failure (33–38). Experimental studies infecting *in vitro* cultured glial cells (including microglia, astrocytes and oligodendrocytes) with COV noted enormous production of inflammatory factors such as IL-6, IL-12, IL-15, and TNF-α (4, 39, 40). Interleukin (IL)-6, is positively correlated with the severity of COVID-2019 symptoms (33–40). Exaggerated immune responses with SARS-COV-2 might contribute to development of acute inflammatory demyelinating poly radiculo-neuropathies.

All the above described mechanisms were not based on studies on peripheral neurons or Schwann cells, therefore the exact mechanism remains unknown. Further dedicated studies are required to undermine the exact cause of neuromuscular invasion of SARS-COV 2 virus.

## Conclusion

Neurological complications with SARS-COV-2 are being reported exponentially. Majority literature is anecdotal and reported in the form of isolated case reports and case series. In order to better understand the causal relationship, and underlying pathophysiology, meta-analysis of these studies is warranted. Physicians must familiarize themselves with the rapidly evolving literature to provide uptodate care to the affected patients.

## Author Contributions

NK, NN, and SA contributed to literature research, manuscript writing. RG made revisions and helped in editing the manuscript.

## Conflict of Interest

The authors declare that the research was conducted in the absence of any commercial or financial relationships that could be construed as a potential conflict of interest.
